# Reticulation pattern without honeycombing on high-resolution CT is associated with the risk of disease progression in interstitial lung diseases

**DOI:** 10.1186/s12890-022-02105-9

**Published:** 2022-08-14

**Authors:** Minna Mononen, Eeva Saari, Hannele Hasala, Hannu-Pekka Kettunen, Sanna Suoranta, Hanna Nurmi, Miia Kärkkäinen, Tuomas Selander, Jukka Randell, Jari Laurikka, Toomas Uibu, Heikki Koskela, Riitta Kaarteenaho, Minna Purokivi

**Affiliations:** 1 Division of Respiratory Medicine, Institute of Clinical Medicine, School of Medicine, Faculty of Health Sciences, University of Eastern Finland, POB 1627, 70211 Kuopio, Finland; 2 Center of Medicine and Clinical Research, Division of Respiratory Medicine, Kuopio University Hospital, POB 100, 70029 Kuopio, Finland; 3 Department of Respiratory Medicine, Tampere University Hospital, POB 2000, 33521 Tampere, Finland; 4 Department of Clinical Radiology, Kuopio University Hospital, POB 100, 70029 Kuopio, Finland; 5 Institute of Clinical Radiology, School of Medicine, Faculty of Health Sciences, University of Eastern Finland, POB 1627, 70211 Kuopio, Finland; 6 Acute Services, Kuopio University Hospital, POB 100, 70029 Kuopio, Finland; 7 Science Services Center, Kuopio University Hospital, POB 100, 70029 Kuopio, Finland; 8 Tampere University Heart Hospital and Finnish Cardiovascular Research Center, Tampere University, 33014 Tampere, Finland; 9 Research Unit of Internal Medicine, University of Oulu, POB 500, 90400 Oulu, Finland; 10 Center of Internal Medicine and Respiratory Medicine and Medical Research Center (MRC) Oulu, Oulu University Hospital, POB 20, 90029 OYS Oulu, Finland

**Keywords:** Disease progression, Interstitial lung disease (ILD), Idiopathic pulmonary fibrosis (IPF), Radiology

## Abstract

**Background:**

The disease course of idiopathic pulmonary fibrosis (IPF) is progressive and occasionally, other types of interstitial lung disease (ILD) may progress similarly to IPF. This study aimed to evaluate risk factors for disease progression within 24 months in patients with various ILDs.

**Methods:**

This prospective study obtained 97 patients with a suspected ILD who underwent a transbronchial lung cryobiopsy. The extent of several high-resolution computed tomography (HRCT) patterns was assessed. Due to the inclusion criteria the study population presented a low extent of honeycombing and definite usual interstitial pneumonia (UIP) pattern on HRCT suggesting an early stage of ILD. Disease progression within 24 months despite treatment was defined as a relative decline of ≥ 10% in forced vital capacity (FVC), or a relative decline in FVC of ≥ 5% and one of the three additional criteria: (1) a decline in diffusion capacity to carbon monoxide (DLCO) ≥ 15%; (2) increased fibrosis on HRCT; (3) progressive symptoms, or progressive symptoms and increased fibrosis on HRCT. The same definition was utilized in patients with IPF and other ILDs. Risk factors for disease progression were evaluated in a multivariable logistic regression model.

**Results:**

Disease progression was revealed in 52% of the patients with ILD, 51% of the patients with IPF, and 53% of the patients with other types of ILD. A high extent of reticulation on HRCT (Odds ratio [OR] 3.11, 95% Confidence interval [CI] 1.21–7.98, *P* = 0.019) and never smoking (OR 3.11, CI 1.12–8.63, *P* = 0.029) were associated with disease progression whereas platelet count (OR 2.06 per 100 units increase, CI 0.96–4.45, *P* = 0.065) did not quite reach statistical significance.

**Conclusion:**

Higher extent of reticulation on HRCT and never smoking appeared to associate with the risk of disease progression within 24 months in ILD patients without honeycombing. Approximately half of the patients with ILD revealed disease progression, and similar proportions were observed in patients with IPF and in other types of ILD.

**Supplementary Information:**

The online version contains supplementary material available at 10.1186/s12890-022-02105-9.

## Background

Idiopathic pulmonary fibrosis (IPF) is usually a progressive disease with dismal prognosis [1], although the disease course may vary and is difficult to predict individually [2, 3]. IPF has been thought as the architype of progressive fibrosis even though recent studies have shown that a significant number of patients survived for over a decade before antifibrotic treatment was available [2, 4]. Occasionally, other types of interstitial lung disease (ILD), such as rheumatoid arthritis associated ILD (RA-ILD), systemic scleroderma associated ILD (SSc-ILD), fibrotic hypersensitivity pneumonia (fHP), and acute fibrinous and organizing pneumonia, may progress similarly to IPF [5]. It has been suggested that rather than a specific ILD diagnosis, the disease course should determine the management of ILDs [6] since patients with a progressive disease course have revealed similar survival rates than patients with IPF [7, 8]. Moreover, an antifibrotic drug nintedanib has been found to be effective also in non-IPF ILD patients with disease progression [9].

Disease progression in ILD has been studied in several studies that have excluded patients with IPF [8, 10–12]. Two recent studies included also patients with IPF within the group of progressive disease course [13, 14]. In the results of the above-mentioned studies, the patients with disease progression were older and had lower baseline pulmonary function tests than the patients with a stable disease [13]. Furthermore, disease progression was independently associated with mortality in the whole cohort and when patients with IPF and other ILDs were analyzed separately [14]. Recent studies revealed that more deterioration on high-resolution computed tomography (HRCT) was observed in the progressive patients treated with pirfenidone than in the stable patients with IPF after the initial forced vital capacity (FVC) decline [15]. Furthermore, the stable IPF patients survived longer than those with a rapid disease progression [16]. In an IPF cluster analysis, progressive patients were divided into three different clusters based on three unique expression patterns of proteins, which were pondered to refer different progressive profiles [17]. In addition, among patients with non-IPF ILDs the survival was shorter in patients with disease progression than in patients with a stable disease course [8, 10].

In the recent years, the research on progressive fibrosis has increased. Still, there is little knowledge of the risk factors for disease progression in patients with ILD. Since the progressive disease course is currently an indicator to consider antifibrotic treatment, we created a multivariable model evaluating the risk factors associated with disease progression in patients with ILD. The aim of this study was to evaluate the proportion of progressive ILD cases and to determine the risk factors associated with disease progression within 24 months. Disease progression was defined similarly in the whole ILD cohort also including patients with IPF.

## Methods

### Patient selection and data collection

Consecutive patients with a suspected ILD and a necessity of a histological investigation were prospectively recruited from Kuopio University Hospital (KUH) and Tampere University Hospital (TAUH) pulmonology clinics between January 2015 and December 2019 to this observational cohort study. A written informed consent was obtained from all participants. Inclusion and exclusion criteria are described in the Additional file 1. Due to inclusion criteria the extent of honeycombing and definite usual interstitial pneumonia (UIP) pattern on HRCT were low suggesting the patients were at an early stage of their ILD. Patient data were collected from the electronic medical records of the hospitals. The diagnoses were concluded in a multidisciplinary meeting according to the contemporary international guidelines [18–20]. The study protocol was approved by the Research Ethics Committee of the Northern Savo Hospital District (statement 80/2014) and Tampere University Hospital (R15149), and the study was conducted in compliance with the Declaration of Helsinki (as revised in 2013). The follow-up of the patients was conducted by the routine clinical practices of each hospital.

### Histological and radiological investigations and questionnaires

**Fig. 1:**
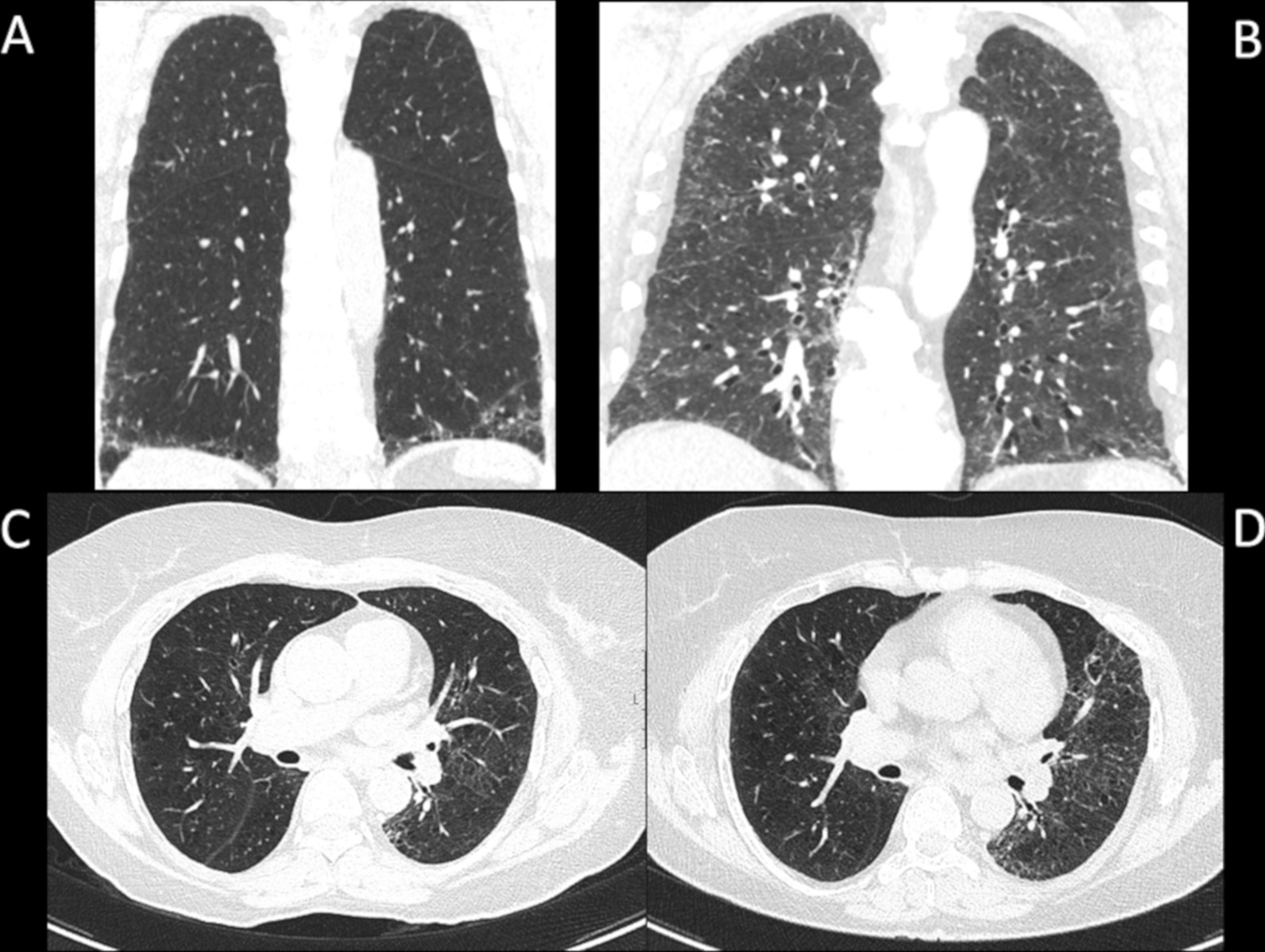
High-resolution computed tomography (HRCT) scans illustrating different reticulation scores. **A** HRCT coronal scan of the whole lung. A patient with reticulation score of the whole lung 6.0, **B** another patient with reticulation score of the whole lung 11.0. **C** HRCT obtained below the level of carina. A third patient with reticulation score of the whole lung 5.0 at the beginning of the study and **D** the same patient with reticulation score of the whole lung 9.0 at 24 months follow-up. This is an original figure created for this manuscript

The 100 study subjects underwent transbronchial lung cryobiopsy and bronchoalveolar lavage (BAL) (see Additional file 1). Before the TBLC, digital volume HRCT scans were available from 84 out of 100 patients. Eleven patients had sequenced HRCT scans. Other comparable CT examinations, such as CT pulmonary angiography, were available from 5 patients. The radiologists evaluated 97% of the images as good quality and 3% as suboptimal quality. The extent of several specific HRCT patterns was assessed separately in three zones of each lung as described previously [21]. The extents of reticulation, honeycombing and ground-glass opacity (GGO) were semi-quantitatively graded on a scale of 0 to 4 (0 = finding absent, 1 = minor peripheral scattered changes, 2 = uniform peripheral or minor central scattered changes, 3 = substantial peripheral changes that penetrated deeply into the lung parenchyma, 4 = very abundant peripheral and central changes) the maximum score being 24. The mean score of the two radiologists for each pattern was used in the analysis. The different reticulation scores and progression of findings on HRCT during follow-up are illustrated in Fig. 1. A more detailed illustration of the scoring has been published previously [21, 22]. Furthermore, the two radiologists agreed on a consensus of the HRCT scans according to the 2018 international statement as a definite UIP, probable UIP, indeterminate with UIP, and alternative diagnosis [19]. The study subjects filled in The University of California, San Diego Shortness of Breath Questionnaire (SOBQ), Leicester Cough Questionnaire (LCQ) and The St George’s Respiratory Questionnaire (SGRQ) at the beginning of the study and in every six months. [23–25].

### The definition of disease progression

Disease progression was defined as a relative decline of ≥ 10% in FVC, or a relative decline in FVC of ≥ 5% and one of the three additional criteria: (1) a decline in diffusion capacity to carbon monoxide (DLCO) ≥ 15%; (2) increased fibrosis on HRCT; (3) progressive symptoms, or progressive symptoms and increased fibrosis on HRCT over 24 months despite treatment [26]. The progressive symptoms were defined as increased (SGRQ, SOBQ) or decreased (LCQ) total scores of a minimum clinically significant difference of the questionnaires [27–29]. The information of progression on HRCT was collected from all the HRCTs available during follow-up. All together 97 patients had sufficient data available at 24 months follow-up.

### Statistical analysis

**Fig. 2:**
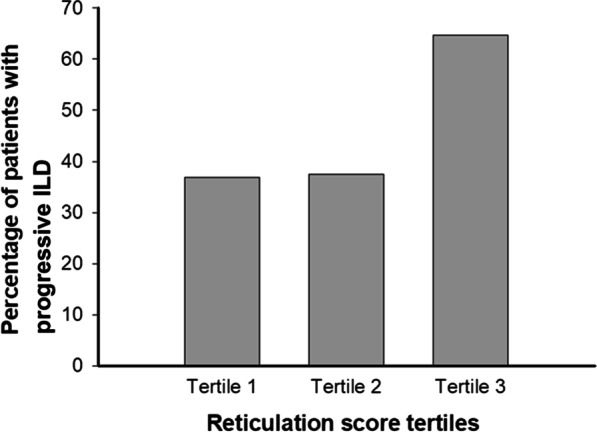
A bar diagram representing the tertiles of reticulation score and the distribution of patients with progressive ILD among these tertiles (Tertile 1 = reticulation score ≤ 6.0, Tertile 2 = reticulation score 6.1–8.9, Tertile 3 = reticulation score ≥ 9.0). ILD = interstitial lung disease. This is an original figure created for this manuscript

Data were expressed as medians and interquartile range (IQR) or frequencies with percentages. Independent-samples *T*-test was used for normally distributed continuous parameters and Mann-Whitney *U* tests for not normally distributed continuous variables, and Chi-square test was used for distribution counts when appropriate. The reticulation score was divided into tertiles which revealed a cut off value of ≥ 9.0 (Fig. 2). Independent variables with a plausible association with the risk of disease progression were selected in the multivariable model using expert evaluation and previous literature of the risk factors for disease progression [30]. Since there were more current or former smokers among the male than the female and higher emphysema score among current or former smokers than the never smokers, gender and emphysema were excluded from the multivariable model. Of the independent variables with strong interrelationships, such as FVC% and DLCO%, SGRQ and SOBQ, and white blood cell count and platelet count, the variable with closer association with disease progression in the bivariable analyses was chosen for the multivariable analysis. A backward stepwise logistic regression model was build using the selected variables (age, smoking status, body mass index [BMI], FVC%, reticulation score, traction bronchiectasis score, platelet count, SOBQ, UIP histology, antifibrotic treatment, and immunosuppressive therapy). The Kaplan-Meier estimator was used to evaluate survival. Inter-observer agreements of the specific HRCT patterns were presented as a kappa (κ) value: good agreement κ = 0.61–0.80, moderate agreement κ = 0.41–0.60 and fair agreement κ = 0.21–0.40. Missing data was excluded. *P* values < 0.05 were considered statistically significant. IBM statistics SPSS software, version 27.0, was used in the statistical analysis.

## Results

### Patient characteristics

Characteristics of the study population are presented in Table 1. Disease progression was revealed in 50 out of 97 patients (52%). There were more former smokers compared to the never smokers in the stable group than in the progressive group. The extent of reticulation on HRCT was higher in the progressive group than in the stable group. The number of immunosuppressive therapies did not differ between the progressive and stable groups (Table 1). Immunosuppressive therapies are presented in detail in the supplementary material (Additional file 1: Table S1). Acute exacerbations were more common in the progressive group than in the stable group (12 vs. 4, *P* = 0.044). Causes of death are presented in the supplementary material (Additional file 1: Table S2). In the subgroup of patients with IPF, 32 out of 63 patients (51%) revealed disease progression within 24 months, and among ILDs other than IPF, 18 out of 34 patients (53%) presented disease progression.

**Table 1:** Characteristics of patients with progressive ILD and a stable disease course

Variable	Progressive ILD (N = 50)	Stable disease (N = 47)	*P* value
Gender (male)	28 (56)	32 (68)	0.221
Age (years)	67.0 (60.8–69.3)	69.0 (60.0–75.0)	0.365
BMI (kg/m^2^)	28.2 (26.4–32.0)	28.1 (25.1–30.7)	0.264
Antifibrotic treatment	19 (38)	16 (34)	0.685
Immunosuppressive therapy^a^	22 (40)	15 (32)	0.221
Never smoker	24 (48)	12 (25)	
Former smoker	18 (36)	30 (64)	0.008*
Current smoker	8 (16)	5 (11)	
Pack years (smokers only)	20.0 (10.0–30.0) 23/26	20.0 (15.0–30.0) 33/35	0.492
FVC %	78.5 (69.5–91.0)	83.0 (72.0–89.0)	0.267
FEV1%	82.0 (72.5–91.0)	83.0 (73.0–91.0)	0.829
DLCO %	61.5 (48.8–73.0)	63.5 (51.4–71.3) 46/47	0.498
White blood cells (10^9^/L)	7.4 (6.0-9.3)	6.9 (5.5–8.1)	0.089
Hemoglobin (g/L)	137.5 (129.5-150.3)	143.0 (134.0-154.0)	0.196
Platelets (10^9^/L)	243.5 (212.3-310.3)	241.0 (199.0-277.0)	0.067
Creatinin (umol/L)	75.0 (64.8–91.3)	76.0 (69.0–91.0)	0.530
GFR (ml/min/1.73m^2^)	84.0 (66.8–93.3)	84.0 (68.5–92.0)	0.847
LCQ total score	16.5 (13.5–19.1)	16.1 (12.4–18.4)	0.411
SGRQ total score	31.9 (14.5–51.7) 46/50	30.9 (16.4–38.4) 43/47	0.837
SOBQ total score	31.5 (7.50–51.0)	22.0 (11.0–40.0)	0.662
Ground-glass opacity score	3.0 (0.6–5.5)	1.5 (0.0-3.8) 41/47	0.105
Reticulation score	10.0 (7.9–12.0)	8.5 (6.0–10.0) 40/47	0.022
Emphysema score	0.0 (0.0–0.0)	0.0 (0.0-2.3) 44/47	0.158
Traction bronchiectasis score	3.5 (2.0-4.6)	2.5 (1.0–4.0) 44/47	0.070
Honeycombing score	0.0 (0.0-1.1)	0.0 (0.0-0.9) 44/47	0.157
UIP histopathology	12 (24)	10 (21)	0.749
UIP pattern on HRCT^b^			0.544
Definite UIP	2 (4)	2 (4)	
Probable UIP	13 (26)	16 (34)	
Indeterminate with UIP	22 (44)	14 (30)	
Alternative diagnosis	13 (26)	15 (32)	
Diagnosis			0.699
Idiopathic pulmonary fibrosis	32 (64)	31 (66)	
Idiopathic nonspecific interstitial pneumonia	9 (18)	5 (11)	
Hypersensitivity pneumonia	5 (10)	5 (11)	
Unclassified ILD	3 (6)	2 (4)	
Respiratory bronchiolitis associated ILD	1 (2)	-	
Connective tissue disease associated ILD	-	1 (2)	
Asbestosis	-	1 (2)	
Cryptogenic organizing pneumonia	-	1 (2)	
Fibrotic sarcoidosis	-	1 (2)	

Numbers are presented as N (%) or median (interquartile range, IQR). BMI = body mass index, DLCO % = diffusion capacity to carbon monoxide, FEV1% = forced expiratory volume in one second percent predicted, FVC % = forced vital capacity percent predicted, GFR = glomerular filtration rate, ILD = interstitial lung disease, LCQ = Leicester cough questionnaire, SGRQ = St George respiratory questionnaire, SOBQ = University of California shortness of breath questionnaire, UIP = usual interstitial pneumonia

*P value between former smoker- never smoker. a. Including the use of oral corticosteroids, azathioprine, or mycophenolate. b. Re-categorized for this study, some of the results have been published previously [31]

### Risk factors for disease progression and survival

**Fig. 3:**
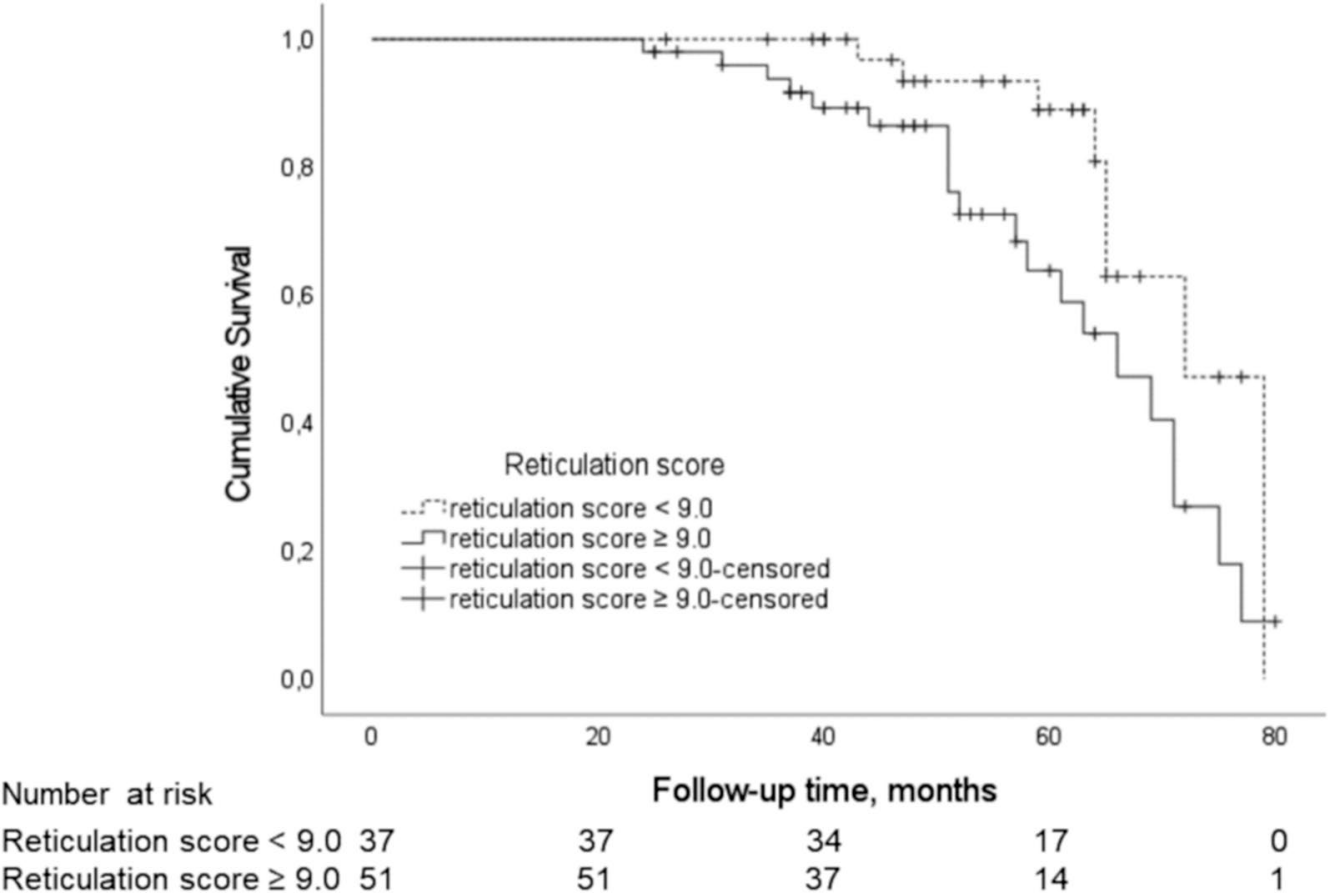
An unadjusted Kaplan-Meier survival curve in ILD patients with higher and lower extent of reticulation on HRCT (Log rank 4.65, *P* = 0.031). Survival curve and P-value represent the time to death or lung transplantation. HRCT = high-resolution computed tomography, ILD = interstitial lung disease. This is an original figure created for this manuscript

**Fig. 4:**
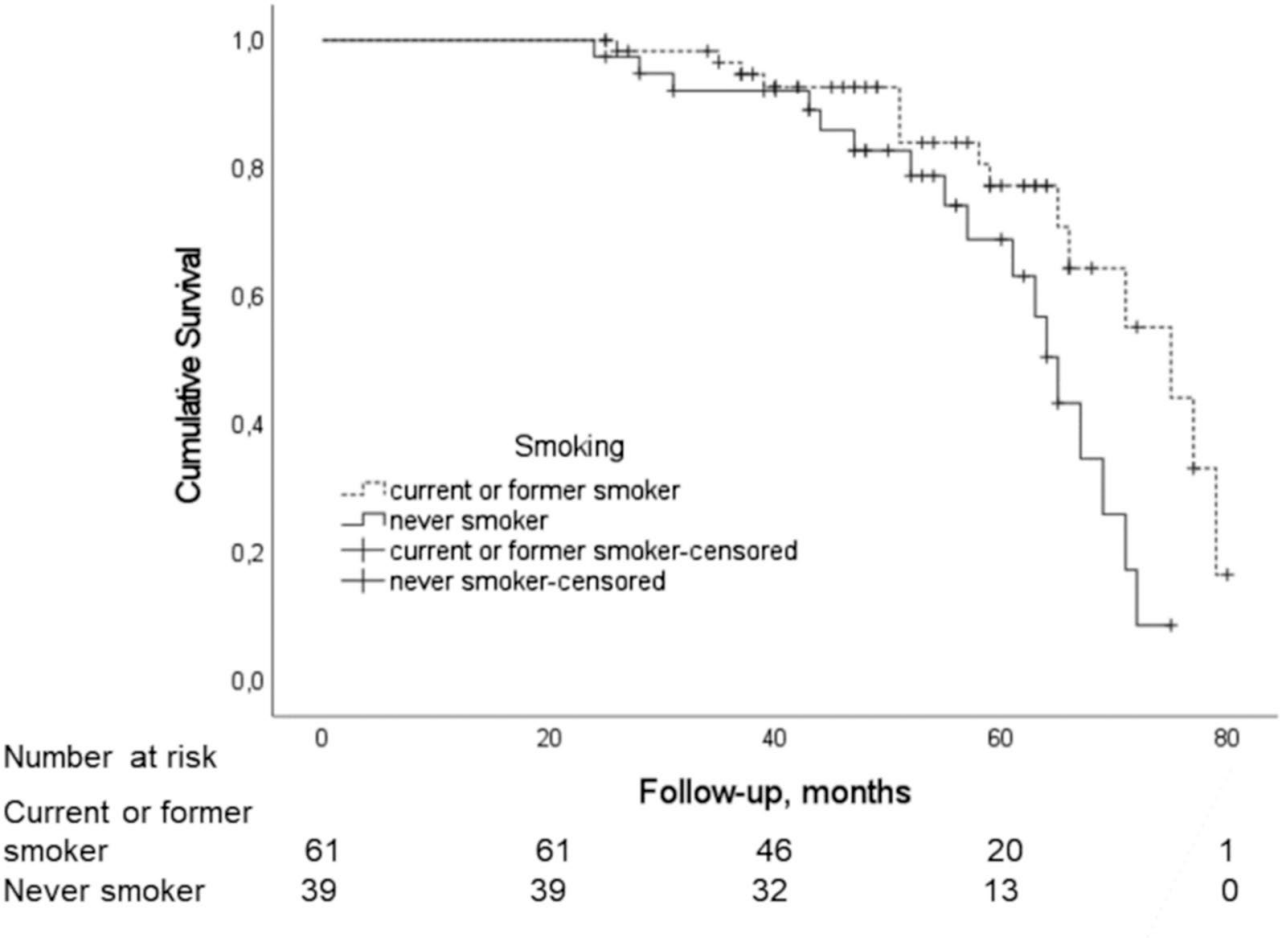
An unadjusted Kaplan-Meier survival curve in ILD patients with different smoking status (Log Rank 5.32, *P* = 0.021). Survival curve and *P* value represent the time to death or lung transplantation. ILD = interstitial lung disease. This is an original figure created for this manuscript

**Fig. 5:**
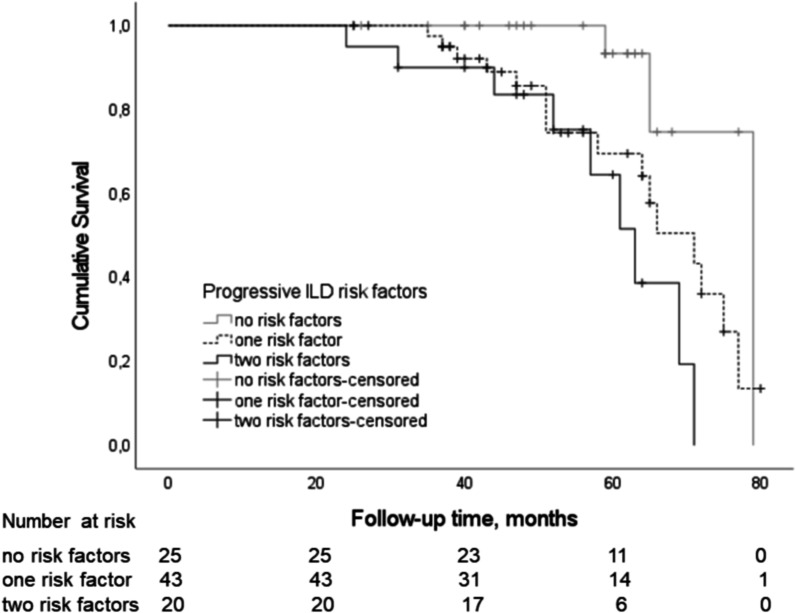
An unadjusted Kaplan-Meier survival curve in patients with risk factors for progressive ILD (Log Rank 9.38, *P* = 0.009). Survival curve and *P* value represent the time to death or lung transplantation. One risk factor = reticulation score ≥ 9.0 on HRCT or never smoking, two risk factors = reticulation score ≥ 9.0 on HRCT and never smoking. HRCT = high-resolution computed tomography, ILD = interstitial lung disease. This is an original figure created for this manuscript

The predictor candidates included in the logistic regression model were age, smoking status, BMI, FVC%, reticulation score, traction bronchiectasis score, SOBQ total score, platelet count, UIP histopathology, antifibrotic treatment, and immunosuppressive therapy. The reticulation score ≥ 9.0 on HRCT and never smoking were associated with the risk of disease progression in ILD whereas platelet count did not quite reach statistical significance (Table 2). The median follow-up time of patients with a stable disease course was 53 months (IQR 41.0–65.0). Patients with higher extent of reticulation on HRCT had shorter median survival (66 months, 95% CI 56.9–75.1) than patients with lower extent of reticulation (72 months, 95% CI 64.6–79.4) (Fig. 3). Never smokers had shorter median survival (65 months, 95% CI 61.6–68.4) than current or former smokers (75 months, 95% CI 65.2–84.8) (Fig. 4). Patients with two risk factors (reticulation score ≥ 9.0 and never smoking) had shorter median survival (63 months, 95% CI 55.5–70.5) than patients with one (71 months, 95% CI 61.3–80.7) or no risk factors (79 months, 95% CI not applicable) (Fig. 5).

**Table 2:** Adjusted^a^ logistic regression analysis for the risk factors of disease progression in patients with ILD

Variable	Odds Ratio	95% Confidence interval	P-value
Reticulation score ≥ 9.0	3.11	1.21–7.98	0.019
Platelet count (per 100 units increase)	2.06	0.96–4.45	0.065
Never smoking	3.11	1.12–8.63	0.029

^a^Includes the following covariates: age, smoking status, body mass index, forced vital capacity percent predicted, reticulation score, traction bronchiectasis score, University of California Shortness of Breath Questionnaire total score, platelet count, usual interstitial pneumonia histopathology, antifibrotic treatment, and immunosuppressive therapy. ILD = interstitial lung disease

### The interobserver agreement

The inter-observer agreement between the two radiologists was moderate to good regarding the different HRCT patterns (ground-glass opacity κ = 0.481, honeycombing κ = 0.655, emphysema κ = 0.697, and traction bronchiectasis κ = 0.440).

## Discussion

In this real-life study we demonstrated that higher extent of reticulation on HRCT and never smoking were associated with the risk of disease progression within 24 months in patients with ILD whereas high platelet count did not quite reach statistical significance. The proportion of patients with disease progression was 52% of the patients with ILD. Furthermore, we observed somewhat unexpectedly that only 51% of patients with IPF presented disease progression regardless of antifibrotic treatment. In addition, we observed that the patients with higher extent of reticulation on HRCT had shorter median survival than patients with lower extent of reticulation as well as never smokers had shorter median survival than current or former smokers. Furthermore, patients with two risk factors for disease progression (reticulation score ≥ 9.0 and never smoking) had shorter median survival than patients with one or no risk factors.

Our study subjects consisted of patients whose HRCT scans did not present a definite UIP pattern, and the extent of honeycombing was minimal, making a histological investigation of lung tissue samples necessary. We observed that higher extent of reticulation in the absence of honeycombing on HRCT was associated with the risk of disease progression in patients with ILD. To the best of our knowledge, this kind of association has not been observed previously, although in several studies, reticulation pattern has been associated with mortality in patients with fibrotic idiopathic interstitial pneumonia (IIP), IPF, and ILD [16, 32–34]. In patients with IPF, visually scored reticulation and higher interstitial HRCT score have shown to be associated with death [16, 33]. In addition, a study of 255 patients with ILD showed that an automated CT analysis of global reticulation volume was associated with mortality [34]. Likewise, a fibrosis score defined as a combination of reticulation and honeycombing has been associated with mortality in patients with ILD other than IPF [35, 36]. An increase of 10% in the extent of fibrosis on HRCT was associated with disease progression in 6 months among IPF patients treated with pirfenidone [15]. In our study, the extent of honeycombing was minimal due to the study protocol which included patients who needed a transbronchial lung cryobiopsy to establish the diagnosis. In a recent study focusing on peripheral blood proteins, patients with progressive ILD other than IPF and a high-risk proteomic signature showed a larger 1-year change in FVC than patients with a low-risk proteomic signature [37]. The difference in FVC change was similar in a subgroup of patients with definite or probable UIP pattern on HRCT [37].

Platelets are associated with the progression, control, and resolution of inflammation but the mechanisms of these processes are poorly understood [38]. Platelet hyperactivity has been observed in patients with IPF compared to controls [39] and a detectable platelet activation was found in the lungs of patients with SSc-ILD but not in patients with SSc without ILD [40]. A recent animal study has demonstrated that in mice with bleomycin-induced pulmonary fibrosis, platelets accumulated into the lung tissue, the platelet number in BAL correlated with fibrotic markers, and that platelet depletion reduced fibrosis and modestly inhibited pulmonary function changes [41]. Thus, our observation of a higher platelet count associating with the risk of disease progression in ILD is in line with the previous studies.

Smoking has been shown to associate with the risk of IPF [42, 43]. However, in several studies current smoking has been associated with a longer survival in patients with IPF [44–46]. Another study of IPF patients showed that current or former smokers had longer survival than never smokers [47]. In patients with autoimmune disease associated ILD, current or former smoking was associated with disease progression and mortality [48] whereas, in a study of patients with RA-ILD, the survival was similar between current or former and never smokers [49]. In our study, never smoking was associated with the risk of disease progression in ILD and never smokers had shorter median survival than current or former smokers. Instead, in recent studies, smoking was not associated with mortality in patients with progressive ILD [14] and an increase in pack years did not associate with the risk of disease progression in patients with ILD in a multivariable model [13]. Thus, the association between smoking and disease progression in ILD merits further investigations.

The proportion of patients with disease progression was similar in our study compared to other recently published studies [13, 14]. Disease progression was presented in 50% of ILD patients in the Canadian study and in 42% in the Japanese study compared to the 51% in our study [13, 14]. The Japanese study used the same definition of disease progression as we did, but they did not include HRCT analysis since they estimated that the proportion of patients who would be defined as progressive due to HRCT changes would be small [14]. The Canadian study had different definition for disease progression but most of the patients (49%) in that study presented FVC decline ≥ 10% as the sign of disease progression [13]. Similarly, in our study most of the patients (68%) presented FVC decline ≥ 10% as the sign of disease progression within 24 months. We observed only 51% of the patients with IPF to have a progressive disease course which could not be explained by the antifibrotic treatment. Similarly, the Canadian and Japanese studies observed that 59% and 59.4% of the patients with IPF revealed disease progression, respectively [13, 14]. The results of the above-mentioned studies and our study showed that not all patients with IPF fulfill the definition of disease progression within 24 months. The proportion of non-IPF ILD patients with disease progression was similar in our study compared to the previous studies despite the variable definitions used to describe disease progression [10, 11].

Previous studies have demonstrated that non-IPF ILD patients with disease progression have worse survival than patients with stable ILD [8, 10]. In addition, it has been demonstrated that non-IPF ILD patients with disease progression have similar survival than patients with IPF [7, 8]. However, Torrisi et al. did not find differences in survival between the progressive ILD and stable ILD in patients with non-IPF ILDs, whereas the survival was longer in patients with disease progression than in patients with IPF [11]. Another study demonstrated a greater three-year survival among patients with IPF (72%) than non-IPF ILD patients with disease progression (64%) [12]. Likewise, in a recent study, the median survival was longer in patients with stable IPF (84.8 months) than in patients with progressive non-IPF (52.3 months) whereas the median survival was shortest in patients with progressive IPF (31.8 months) [14].

We demonstrated a shorter median survival in patients with risk factors for disease progression observed in the present study than in patients with no risk factors. Different categorization, different amounts of specific ILD types, and a different proportion of UIP pattern on HRCT may explain the differences in the survival results. However, a similar survival has been demonstrated between the different disease progression definitions used in non-IPF patients [10]. The majority of our study population constituted of patients with IPF and none of the patients had a definite UIP pattern on HRCT. Furthermore, the number of patients with connective tissue associated ILD (CTD-ILD) (n = 1) in our study was minimal since the role of lung biopsy is small in the diagnosis of CTD-ILDs. Kwon et al. and Chen et al. included mostly patients with autoimmune disease associated ILD or CTD-ILD (67.7% and 38.6% respectively) when the UIP pattern was observed in 45.7% and 38.6% of patients, respectively [8, 10]. Whereas most of the patients in the study of Torrisi et al. were diagnosed with fHP (35.5%) and the proportion of UIP pattern on HRCT was not described [11].

The small number of patients can be regarded as a limitation of this study. The requirement of a histological investigation and an exclusion of patients with severe pulmonary function impairment could have caused selection bias in the study population. In addition, the multivariable model was not validated. For this reason, the results should be considered as observational rather than representing causality. Furthermore, nearly 80% of the study population constituted of patients with IIP, which should be taken into consideration when assessing the generalizability of the results. However, this study population included well characterized patients with ILD who underwent histopathological investigation and whose HRCTs were reanalyzed, and HRCT patterns were quantified. Moreover, the symptoms were evaluated systematically by questionnaires, and furthermore, laboratory tests and lung function tests were available.

## Conclusion

Reticulation on HRCT was associated with the risk of disease progression within 24 months in patients with ILD not revealing a definite UIP pattern on HRCT. Approximately half of the patients presented a progressive disease course. Similarly, in a subgroup of patients with IPF 51% presented disease progression within 24 months. The results reinforce the role of HRCT in the evaluation of disease progression among patients with ILD. Furthermore, the results support the evidence that the disease progression of IPF is not as uniform as it has been assumed.

## Supplementary Information

**Additional file 1.** Describing the inclusion and exclusion criteria and the transbronchial lung cryobiopsy protocol in detail as well as including. **Tables S1–2** Describing immunosuppressive therapiesand causes of death in detail.

## Data Availability

The datasets generated and analyzed during the current study are not publicly available due the gathering of the data is continuing but are available from the corresponding author on reasonable request.

## Abbreviations

BAL: Bronchoalveolar lavage
BMI: Body mass index
CI: Confidence interval
CTD-ILD: Connective tissue disease associated interstitial lung disease
DLCO: Diffusion capacity to carbon monoxide
fHP: Fibrotic hypersensitivity pneumonia
FVC: Forced vital capacity
GGO: Ground-glass opacity
HRCT: High-resolution computed tomography
IIP: Idiopathic interstitial pneumonia
ILD: Interstitial lung disease
IPF: Idiopathic pulmonary fibrosis
IQR: Interquartile range
KUH: Kuopio University Hospital
LCQ: Leicester Cough Questionnaire
OR: Odds ratio
RA-ILD: Rheumatoid arthritis associated interstitial lung disease
SGRQ: St George Respiratory Questionnaire
SOBQ: University of California, San Diego Shortness of Breath Questionnaire
SSc-ILD: Systemic scleroderma associated interstitial lung disease
TAUH: Tampere University Hospital
TBLC: Transbronchial lung cryobiopsy
UIP: Usual interstitial pneumonia
