# Supplementary material for: Reticulation pattern without honeycombing on high-resolution CT is associated with the risk of disease progression in interstitial lung diseases
Source: BMC Pulm Med. 2022 Aug 14;22:313. doi: 10.1186/s12890-022-02105-9 (PMC9375921; doi:10.1186/s12890-022-02105-9)
Supplement: Supplementary file 1 — Additional file 1. Describing the inclusion and exclusion criteria and the transbronchial lung cryobiopsy protocol in detail as well as including. Tables S1–2 Describing immunosuppressive therapiesand causes of death in detail. [file 12890_2022_2105_MOESM1_ESM.docx]

Supplementary material, Additional file 1.

Inclusion and exclusion criteria and the transbronchial lung cryobiopsy protocol

Inclusion criteria: a referral to a tertiary university hospital (Kuopio University Hospital [KUH] or Tampere University Hospital [TAUH]) for a suspected interstitial lung disease and a requirement of a histological investigation in the diagnosis of interstitial lung disease.

Exclusion criteria: acute myocardial infarct, acute and untreated heart disease, active tuberculosis, anticoagulation treatment that could not be withheld, abnormal bleeding history or bleeding parameters (thrombocytes, international normalized ratio [INR], activated partial thromboplastin time [APTT]), forced expiratory volume in one second (FEV1) < 50 %, total lung capacity (TLC) < 50 %, diffusion capacity to carbon monoxide (DLCO) < 50 %, mean pulmonary artery pressure > 55 mmHg in echocardiogram (ECHO), irreparable hypoxia (arterial pressure of oxygen [pO_2_] < 8 kPa). Patients with body mass index (BMI) > 30 kg/m^2^ were excluded if the mean pulmonary artery pressure in ECHO was > 55 mmHg. Anticoagulants and antiplatelets (except for acetyl salicylic acid [ASA]) were withheld at the time of biopsy.

All the study subjects had transbronchial lung cryobiopsy (TBLC) and bronchoalveolar lavage (BAL) performed in an operating room in an outpatient setting. The TBLC was conducted on intubated patients in general anesthesia using mechanical ventilation. A flexible therapeutic bronchoscope with a 2.8 mm working channel and a 6.2 mm outer diameter (Olympus BF-1T180 Evis Exera II) was used to obtain BAL. Preferred biopsy site was previously decided by the operator according to the CT findings. A deflated balloon blocker (Fogarty® balloon) was inserted through the endotracheal tube’s side port just above the preferred biopsy site and a multiple use cryoprobe (KUH 2.4 mm; TAUH 1.9 mm; ERBE, Tübingen, Germany) through the operating channel of the flexible bronchoscope. Fluoroscopy guidance was used to reassure 1-2 cm distance from the pleura. The cryoprobe was cooled for approximately 5 seconds and the sample was extracted along with the bronchoscope and thawed in saline at a room temperature. Simultaneously, the balloon blocker was inflated prophylactically to prevent bleeding. The blocker remained inflated until re-insertion of the bronchoscope. In the absence of bleeding the blocker was slowly deflated. Up to six biopsies (aim of 5 biopsies [mean 5, range 1-6]) were obtained in different segments of one or two lobes.

Supplementary Table S1. Immunosuppressive therapies in patients with interstitial lung diseases

| Therapy | Progressive ILD (N = 50) | Stable ILD (N = 47) |
| --- | --- | --- |
| Oral corticosteroids | 22 (40) | 14 (30) |
| Azathioprine | 4 (8) | 3 (6) |
| Mycophenolate | 2 (4) | - |

Numbers are presented as N (%). ILD = interstitial lung disease

Supplementary Table S2. Causes of death in patients with interstitial lung diseases

| Progressive ILD | Stable ILD |
| --- | --- |
| Idiopathic pulmonary fibrosis (11) | Gastrointestinal cancer (4) |
| Nonspecific interstitial pneumonia (4) | Idiopathic pulmonary fibrosis (1) |
| Cardiovascular disease (2) | Nonspecific interstitial pneumonia (1) |
| Lung cancer (1) | Lung cancer (1) |
| Alcohol use induced epileptic seizure (1) | Leukemia (1) |
| Nonspecific fibrosis, diffuse alveolar damage (1) | Cardiovascular disease (1) |
|  | Bowl occlusion (1) |

Numbers are presented as (N). ILD = interstitial lung disease
